# The association between adolescent interoceptive awareness and future exercise possible self: a mediation model

**DOI:** 10.7717/peerj.21780

**Published:** 2026-10-12

**Authors:** Jing Huang, Donglin Hu, Peng Wu, Siyu Tian, Pingping Xie, Yang Pan

**Affiliations:** 1 School of Wushu and National Traditional Sports, Tianjin University of Sport, Tianjin, China; 2 College of Sports Industry and Leisure, Nanjing Sport Institute, Nanjing, China; 3 School of Sport Education, Tianjin University of Sport, Tianjin, China; 4 Department of Physical Education, Tianjin Medical University, Tianjin, China

**Keywords:** Interoceptive awareness, Self-concept clarity, Athletic engagement, Future exercise possible self, Adolescents

## Abstract

**Objective:**

Based on the perspective of embodied cognition, this study examined the association between interoceptive awareness and future exercise possible self among adolescents, and investigated the mediating roles of self-concept clarity and athletic engagement in this association, as well as a potential chain mediation pathway.

**Methods:**

A stratified random sampling approach was adopted to recruit 1,222 students from Grades 7 to 12 across six major regions of China in May 2025, and 1,131 retained for analysis (55.9% boys, 44.1% girls). Participants completed the Multidimensional Assessment of Interoceptive Awareness (MAIA-2), the Self-Concept Clarity Scale (SCCS), the Athlete Engagement Questionnaire (AEQ), and the Future Exercise Possible Self Inventory (FEPSI). Descriptive statistics, correlation coefficients, and regression analyses were performed using SPSS 24.0 to examine the relationships between each dimension of interoceptive awareness and future exercise possible self. Based on the significance of regression coefficients, a chain mediation structural equation model was constructed in Amos 26.0 to explore the comprehensive theoretical framework, with a specific focus on the mediating roles of self-concept clarity and athletic engagement. Bootstrap resampling (5,000 resamples) was conducted to estimate total, direct, and indirect effects.

**Results:**

Interoceptive awareness and its dimensions were positively correlated with self-concept clarity, athletic engagement, and future exercise possible self (*p* < 0.01). After controlling for demographic variables, seven of the eight interoceptive awareness dimensions significantly predicted future exercise possible self (*p* < 0.05), with the “body listening” dimension being the only exception. The structural model yielded excellent fit indices (*χ*^2^/df = 1.171, CFI = 0.992, Root Mean Square Error of Approximation (RMSEA) = 0.012). Interoceptive awareness exerted a significant total effect on future exercise possible self (β = 0.586), including a direct effect (β = 0.329) and an indirect effect (β = 0.257). Self-concept clarity (β = 0.044, *p* < 0.05) and athletic engagement (β = 0.195, *p* < 0.01) independently mediated this relationship. Furthermore, they jointly formed a significant chain mediation pathway (β = 0.018, *p* < 0.01).

**Conclusions:**

Interoceptive awareness has a cross-sectional positive association with adolescents’ future exercise possible self, both directly and indirectly through enhanced self-concept clarity and elevated athletic engagement. These findings delineate a sequential pattern linking bodily awareness, self-knowledge integration, motivational experiences, and future-oriented exercise identity. Cultivating adolescents’ sensitivity to and positive interpretation of internal bodily signals, together with strengthening self-concept clarity and athletic engagement, may facilitate the development of positive exercise-related self-perceptions and long-term physical activity beliefs.

## Introduction

### Future exercise possible self in adolescents

The concept of possible selves refers to individuals’ cognitive representations of their potential future selves, which acts as a critical psychological mechanism connecting current states to future goals (Markus & Nurius, 1986). Adolescence is a pivotal developmental stage characterized by growing self-awareness and ongoing identity formation (Becht et al., 2021). During this period, individuals actively construct their possible selves and systematically evaluate their capabilities (Hards et al., 2024). By integrating personal goals, values, and past experiences, the envisioned possible selves act as a motivational catalyst and significantly shape behavioural decisions (Oyserman et al., 2004). This phenomenon is particularly prominent in adolescence, a phase marked by remarkable progress in cognitive abilities, emotional regulation, and social adaptability (Vetter et al., 2013).

A large amount of research has demonstrated that adolescent participation in physical activities not only promotes physical and mental well-being but also significantly improves academic performance and social adaptability (Somerset & Hoare, 2018). In the domain of sport and exercise, the future exercise possible self includes both the aspirational “exercise self one hopes to become” and the aversive “exercise self one hopes to avoid”. A positive future exercise possible self provides constructive behavioral guidance for adolescents, enhances their intrinsic motivation for sports participation, fosters a stronger sense of sports self-efficacy, and offers clear cognitive guidance and behavioral support to sustain physical activity engagement, thereby contributing to the establishment of a clear and positive long-term sports identity (Choudhury et al., 2024). Given that adolescence is a critical period for shaping exercise attitudes, forming sports-related identities, and establishing lifelong exercise habits, exploring the psychological correlates of adolescents’ future exercise possible self holds great theoretical and practical significance.

### Interoceptive awareness and future exercise possible self

Interoceptive awareness is defined as an individual’s ability to perceive, interpret, and utilize internal bodily signals such as heart rate, respiration, and muscle tension (Wang et al., 2025). From the perspective of embodied cognition, interoceptive awareness serves as a fundamental interface between bodily sensations and cognitive processes, contributing to self-evaluation, perceived competence, and expectations of future capabilities (Chen, 2022). Previous research has confirmed that athletes generally exhibit higher levels of interoceptive awareness (Seabury, Benton & Young, 2023), which facilitates the use of more effective sports strategies and thus improves athletic performance (Maruo, 2025). Meanwhile, interoceptive awareness regulates an individual’s experiences of positive and negative stress during exercise and competitions (di Fronso et al., 2022) and plays a key role in reducing the risk of sports injuries (Iona et al., 2022). It can be inferred that a high level of interoceptive awareness supports the physiological and psychological development required for adolescents to develop advanced athletic skills and enhance confidence in sports. During physical activity, real-time physiological feedback helps adolescents recognize their physical limits and potential, which in turn shapes their self-perceptions of future exercise capacity and developmental trajectories. Therefore, interoceptive awareness serves not only as a basic component of bodily signal processing but also as an important psychological starting point for the formation of future exercise possible selves. From this perspective, the way adolescents perceive and interpret bodily signals is closely related to how they envision and plan their future physical activity participation.

Furthermore, to clarify how physiological processes are transformed into a coherent future self-concept, it is essential to examine this connection across multiple levels of psychological organization. Self-concept clarity and athletic engagement provide a framework for understanding this transformation.

### The mediating role of self-concept clarity

Self-concept clarity refers to the degree to which self-knowledge is clearly defined, internally consistent, and temporally stable (Campbell, Trapnell & Heine, 1996). According to the self-verification theory, individuals tend to maintain a coherent and stable self-concept (Briñol & Petty, 2022). As a continuous and autonomous “internal information-gathering system”, interoceptive awareness provides authentic and stable bodily cues that support the construction and refinement of self-schemas (Garfinkel et al., 2015). When adolescents accurately perceive physiological changes during exercise, such as elevated heart rate, muscle tension, fatigue, or feelings of strength, these bodily cues help them form a clearer understanding of their physical abilities and potential, thereby enhancing self-consistency and confidence (Tian & Sun, 2023). Therefore, self-concept clarity may serve as a key mediator in the relationship between interoceptive awareness and adolescents’ future exercise possible self.

### The mediating role of athletic engagement

Athletic engagement refers to the positive psychological states experienced during sport participation, including vigor, dedication, confidence, and enthusiasm (Ye et al., 2016). Both embodied cognition and self-determination theory suggest that positive bodily signals and emotional experiences can enhance the satisfaction of basic psychological needs such as competence, autonomy, and relatedness (Chen, 2022; Flannery, 2017), thereby promoting sustained motivation and engagement in physical activity. Adolescents with higher interoceptive sensitivity are more likely to associate exercise experiences with enjoyment, perceived control, and competence, leading to higher levels of sustained engagement and effort (Fazekas et al., 2022; Lonsdale, Hodge & Raedeke, 2007). These accumulated positive experiences help adolescents develop more positive beliefs about their future athletic abilities and strengthen their future exercise possible self (Guo, Qi & Yang, 2021). Accordingly, athletic engagement may represent another important psychological pathway linking interoceptive awareness to future exercise possible self. In other words, the transformation of bodily sensations into positive exercise experiences plays a key role in this association.

### Self-concept clarity and athletic engagement

Self-concept clarity and athletic engagement reinforce each other. A clear and stable self-schema provides psychological resources to support sustained physical activity engagement, while continuous engagement strengthens positive self-perceptions over time (Chen, Li & Ye, 2024; Wu et al., 2024). Consequently, the sequential pathway, that is, interoceptive awareness → self-concept clarity → athletic engagement → future exercise possible self, constitutes a continuous psychological process, reflecting the developmental progression from bodily awareness to self-understanding and finally to behavioral investment. Collectively, these processes contribute to the formation of a more positive future exercise possible self.

### The present study

Existing research has not systematically examined the continuous psychological mechanism of “body signal processing → self-structure → motivation investment → future self”. Although the prevailing theoretical framework suggests that interoceptive awareness may influence adolescents’ future-oriented exercise self-perceptions, empirical studies rigorously investigating this relationship remain scarce. Importantly, no prior research has explicitly verified the links between interoceptive awareness, self-concept clarity, athletic engagement, and future exercise possible self. This gap limits a comprehensive understanding of the mechanism through which interoceptive awareness affects future exercise possible self. To address this gap, this study draws on existing literature and proposes the following hypotheses:

H1: Interoceptive awareness is positively correlated with future exercise possible self.

H2: Self-concept clarity mediates the association between interoceptive awareness and future exercise possible self.

H3: Athletic engagement mediates the association between interoceptive awareness and future exercise possible self.

H4: Self-concept clarity and athletic engagement play a chain mediating role in the

relationship between interoceptive awareness and future exercise possible self.

This study makes several key contributions to the literature:

(1) It is the first study to integrate interoceptive awareness into the research framework of future exercise possible self, applying the perspective of embodied cognition to explain the developmental mechanisms of future exercise self-perceptions.

(2) It proposes and empirically examines the mediating roles of self-concept clarity and athletic engagement in the relationship between interoceptive awareness and future exercise possible self, providing structured evidence for the sequential psychological processes involved.

(3) It uses a large, multi-regional adolescent sample (*N* = 1,131) covering various geographic regions, grade levels, and school types, which improves the generalizability and external validity of the findings and provides important empirical evidence for the development of adolescent exercise psychology.

## Materials and Methods

### Participants

In May 2025, a stratified random sampling strategy was used to recruit students in Grades 7–12 from six major regions of China (North, East, South, Central, Southwest, and Northeast). A total of 1,222 electronic questionnaires were distributed to students *via* school principals and teachers. After excluding 91 invalid questionnaires (due to careless responses, inconsistent responses to reverse-coded items, or incomplete data), a total of 1,131 valid responses were obtained. The sample included 632 boys (55.9%) and 499 girls (44.1%). The grade distribution was as follows: Grade 7 (191 students, 16.9%), Grade 8 (366 students, 32.4%), Grade 9 (116 students, 10.3%), Grade 10 (109 students, 9.6%), Grade 11 (163 students, 14.4%), and Grade 12 (186 students, 16.4%). A total of 787 students (69.6%) were from urban areas and 344 students (30.4%) from rural or township areas. In addition, 856 students (75.7%) attended public schools and 275 students (24.3%) attended private schools. The regional distribution was as follows: North China (98 students, 8.7%), East China (708 students, 62.6%), South China (100 students, 8.8%), Central China (62 students, 5.5%), Southwest China (116 students, 10.3%), and Northeast China (47 students, 4.2%). In terms of sports proficiency, 393 students (34.7%) had no athletic grade, 681 students (60.2%) were third-level athletes, 43 students (3.8%) were second-level athletes, and 14 students (1.2%) were first-level athletes. In terms of training duration, 29 students (2.56%) had ≦ 1 year of training, 256 students (22.6%) had 1–2 years of training habit, 240 students (21.2%) had 3–4 years of training habit, 333 students (29.4%) had 5–6 years of training, and 273 students (24.13%) had more than 6 years of training.

Sample size calculation using G*Power software indicated that a minimum of 107 participants was required for this study, and the actual sample size met this requirement. Proportional quotas for region, gender, grade, and school type were preset and controlled during sampling to ensure sample representativeness.

### Inclusion criteria

(1) Aged 11–18 years;

(2) Currently enrolled in school;

(3) Capable of independently understanding and completing the questionnaire.

### Exclusion criteria

(1) Severe psychological or physical conditions affecting regular physical activity;

(2) Participation in sports camps or intensive competitions in the past month to avoid short-term fluctuations in physical activity behavior.

All participants voluntarily participated in the study, and written informed consent was obtained from parents or legal guardians for minor participants. Ethical approval was granted by the Ethics Committee of Tianjin University of Sport (Approval No. TJUS2025-080), and all procedures were performed in accordance with the Declaration of Helsinki.

### Measures

#### Demographic questionnaire

Demographic variables included region, sex, grade, sports proficiency, years of training, residential area, and school type. All information was self-reported by participants.

#### Interoceptive awareness (Mehling et al., 2012)

Interoceptive awareness was measured using the 37-item Multidimensional Assessment of Interoceptive Awareness (MAIA-2), which comprises eight subscales: noticing, not-distracting, not-worrying, attention regulation, emotional awareness, self-regulation, body listening, and trust. Items were rated on a 6-point Likert scale (0 = never to 5 = always). Items 5, 6, 7, 8, 9, 10, 11, 12, and 15 were reverse-coded. Specific items are listed in the supplementary codebook. The reliability and validity of the MAIA-2 have been verified in the cultural context of this study’s sample (Wang et al., 2025; Jones et al., 2021). In this study, Cronbach’s α for the total scale was 0.960, and for subscales ranged from 0.823 to 0.921, indicating good internal consistency.

#### Self-concept clarity (Niu et al., 2016)

The Self-Concept Clarity Scale (SCCS) contains 12 items, rated on a 5-point Likert scale (1 = strongly disagree to 5 = strongly agree). Items 6 and 11 were positively phrased, and the rest were reverse-coded. Specific items are provided in the supplementary codebook. This scale has been validated in the cultural context of the study sample (Tian & Sun, 2023). In this study, Cronbach’s α was 0.950, indicating high internal consistency.

#### Athletic engagement (Ye et al., 2016)

Athletic engagement was assessed with the 16-item Athlete Engagement Questionnaire (AEQ), which includes four subscales: confidence, vigor, dedication, and enthusiasm. Items were rated on a 5-point Likert scale (1 = never to 5 = always). Specific items are listed in the supplementary codebook. The scale has established reliability and validity in the study’s cultural context (Tian & Sun, 2023). In this study, Cronbach’s α for the total scale was 0.909, with subscale coefficients of 0.865 (confidence), 0.875 (vigor), 0.876 (dedication), and 0.865 (enthusiasm).

#### Future exercise possible self inventory (Zhang & Mao, 2004)

The Future Exercise Possible Self Inventory (FEPSI) includes 5 items rated on a 7-point Likert scale (1 = not at all true of me to 7 = very true of me). Detailed items are available in the supplementary codebook. This scale has shown good reliability and validity in the study sample (Zhang & Mao, 2004). In this study, Cronbach’s α was 0.898.

### Statistical analyses

The data preprocessing was performed using Microsoft Excel. Descriptive statistics (median and interquartile range) and Spearman correlation analyses were conducted in SPSS 24.0 (IBM Corp., Armonk, NY, USA). Regression analyses were also performed in SPSS 24.0 to examine the associations between the eight dimensions of interoceptive awareness and future exercise possible self, as well as the unique contribution of each dimension. Subsequently, a structural equation model was applied to test the comprehensive theoretical framework. Specifically, a chain mediation structural equation model was constructed in Amos 26.0, with interoceptive awareness as the predictor, self-concept clarity and athletic engagement as mediators, and future exercise possible self as the outcome. A significant regression coefficient between the independent and dependent variables is a prerequisite for mediation analysis (Wen & Ye, 2014). To ensure model rationality and validity, the latent structure of the independent variables was constructed as a second-order factor based only on the statistically significant dimensions of interoceptive awareness identified in regression analysis. Model fit indices were evaluated, and indirect effects were tested using a bootstrap procedure with 5,000 samples and 95% bias-corrected confidence intervals (CIs). Effects were considered statistically significant if the CIs did not include zero. The significance level was set at α = 0.05.

## Results

### Common method bias test

Common method bias was examined using Harman’s single-factor test. Unrotated exploratory factor analysis extracted 14 factors with eigenvalues greater than 1, and the first factor accounted for 32.94% of the variance, which was below the 40% threshold. These results reveal that common method bias did not significantly affect the findings.

### Descriptive statistics and correlation analysis

Median, interquartile range, and correlation matrix for all variables are presented in Table 1. Interoceptive awareness and its dimensions were significantly and positively correlated with self-concept clarity, athletic engagement and its subdimensions, and future exercise possible self (*p* < 0.01). These correlations provide foundational support for the mediation pathways in the structural equation model.

**Table 1:** Descriptive statistics and correlation matrix of study variables.

	**1**	**2**	**3**	**4**	**5**	**6**	**7**	**8**	**9**	**10**	**11**	**12**	**13**	**14**	**15**	**16**
**1**	1.000															
**2**	0.700^**^	1.000														
**3**	0.772^**^	0.524^**^	1.000													
**4**	0.738^**^	0.555^**^	0.569^**^	1.000												
**5**	0.789^**^	0.520^**^	0.538^**^	0.544^**^	1.000											
**6**	0.723^**^	0.535^**^	0.548^**^	0.541^**^	0.516^**^	1.000										
**7**	0.695^**^	0.528^**^	0.535^**^	0.524^**^	0.512^**^	0.509^**^	1.000									
**8**	0.693^**^	0.535^**^	0.549^**^	0.545^**^	0.527^**^	0.508^**^	0.518^**^	1.000								
**9**	0.641^**^	0.490^**^	0.519^**^	0.476^**^	0.514^**^	0.477^**^	0.493^**^	0.498^**^	1.000							
**10**	0.442^**^	0.385^**^	0.399^**^	0.394^**^	0.400^**^	0.385^**^	0.395^**^	0.412^**^	0.355^**^	1.000						
**11**	0.575^**^	0.519^**^	0.535^**^	0.526^**^	0.489^**^	0.526^**^	0.483^**^	0.500^**^	0.470^**^	0.445^**^	1.000					
**12**	0.486^**^	0.440^**^	0.466^**^	0.448^**^	0.411^**^	0.429^**^	0.421^**^	0.435^**^	0.373^**^	0.403^**^	0.773^**^	1.000				
**13**	0.506^**^	0.454^**^	0.453^**^	0.474^**^	0.440^**^	0.438^**^	0.409^**^	0.420^**^	0.424^**^	0.387^**^	0.787^**^	0.510^**^	1.000			
**14**	0.442^**^	0.429^**^	0.425^**^	0.396^**^	0.377^**^	0.408^**^	0.406^**^	0.398^**^	0.366^**^	0.382^**^	0.760^**^	0.498^**^	0.482^**^	1.000		
**15**	0.489^**^	0.401^**^	0.436^**^	0.432^**^	0.420^**^	0.466^**^	0.390^**^	0.424^**^	0.408^**^	0.365^**^	0.769^**^	0.480^**^	0.516^**^	0.468^**^	1.000	
**16**	0.441^**^	0.391^**^	0.390^**^	0.390^**^	0.391^**^	0.394^**^	0.407^**^	0.351^**^	0.390^**^	0.366^**^	0.454^**^	0.360^**^	0.403^**^	0.384^**^	0.380^**^	1.000
**Me**	2.135	2.750	2.667	2.800	2.857	2.600	2.750	2.667	2.667	39.000	50.000	14.000	15.000	15.000	15.000	22.000
**IQR**	1.946	2.500	2.333	2.400	2.286	2.400	2.250	2.333	2.333	25.000	27.000	9.000	9.000	9.000	9.000	11.000

**Notes.**

1 = Interoceptive awareness (total score); 2 = Noticing; 3 = Not-Distracting; 4 = Not-Worrying; 5 = Attention Regulation; 6 = Emotional Awareness; 7 = Self-Regulation; 8 = Body Listening; 9 = Trust; 10 = Self-Concept Clarity; 11 = Athletic Engagement (total score); 12 = Confidence; 13 = Vigor; 14 = Dedication; 15 = Enthusiasm; 16 = Future Exercise Possible Self.

** *p* < 0.01.

### Regression analysis

A series of linear regression analyses were conducted with the eight dimensions of interoceptive awareness (noticing, not-distracting, not-worrying, attention regulation, emotional awareness, self-regulation, body listening, and trust) as predictors and future exercise possible self as the outcome, controlling for demographic variables. Results showed that seven dimensions (excluding body listening) were significantly associated with future exercise possible self. Variance inflation factors (VIF < 5) indicated no substantial multicollinearity among predictors. Detailed results are shown in Table 2.

**Table 2:** Regression analysis of interoceptive awareness predicting future exercise possible self.

**Variable**	**B (β)**	**SE**	**t**	**Tolerance**	**VIF**
Constant	13.246	2.113	6.268^***^	—	—
Region	−0.052 (−0.010)	0.139	−0.292	0.990	1.010
Sex	0.158 (0.011)	0.361	0.441	0.976	1.024
Grade	0.079 (0.020)	0.108	0.732	0.863	1.158
Sport proficiency level	0.326 (0.028)	0.391	0.829	0.578	1.730
Years of training	−0.052 (−0.009)	0.193	−0.270	0.629	1.589
Place of residence	−0.655 (−0.043)	0.387	−1.689	0.989	1.011
School type	−0.563 (−0.035)	0.415	−1.364	0.990	1.010
Noticing	0.478 (0.095)	0.174	2.747^**^	0.539	1.856
Not-Distracting	0.388 (0.076)	0.181	2.149^*^	0.517	1.936
Not-Worrying	0.377 (0.074)	0.179	2.109^*^	0.520	1.922
Attention Regulation	0.495 (0.095)	0.178	2.779^**^	0.553	1.808
Emotional Awareness	0.520 (0.103)	0.173	3.006^**^	0.549	1.821
Self-Regulation	0.612 (0.121)	0.172	3.565^***^	0.564	1.773
Body Listening	0.045 (0.009)	0.172	0.256	0.541	1.847
Trust	0.578 (0.115)	0.164	3.512^***^	0.604	1.655
	**Model fit indices**				
R^2^	0.278				
Adjusted R^2^	0.269				
F	28.664^***^				

**Notes.**

B = unstandardized coefficient; β = standardized coefficient.

* *p* < 0.05.

** *p* < 0.01.

*** *p* < 0.001.

### Mediation model analysis and testing

Based on correlation and regression results, a second-order structural equation model was constructed with interoceptive awareness as the predictor, self-concept clarity and athletic engagement as mediators, and future exercise possible self as the outcome (Fig. 1). The model exhibited excellent fit to the data:

**Figure 1:**
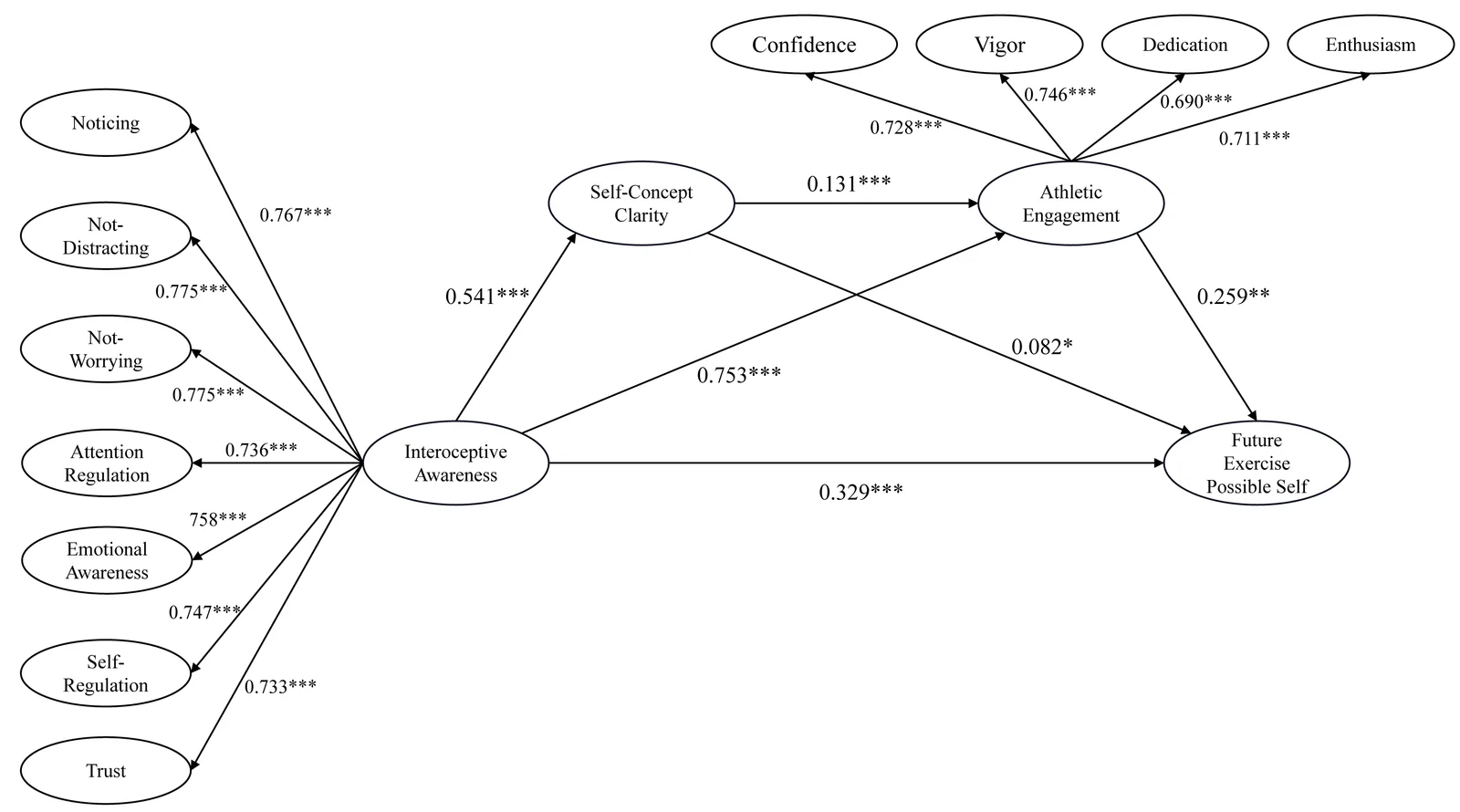
Second-order structural equation model linking interoceptive awareness to future exercise possible self *via* self-concept clarity and athletic engagement. Notes: *p < 0.05, **p < 0.01, ***p < 0.001.

*χ*^2^ = 2490.793, *p* < 0.001, *df* = 2127, *χ*^2^/*df* = 1.171, Goodness-of-Fit Index (GFI) = 0.939, Adjusted Goodness-of-Fit Index (AGFI) = 0.935, Normed Fit Index (NFI) = 0.950, Incremental Fit Index (IFI) = 0.992, Comparative Fit Index (CFI) = 0.992, Tucker-Lewis Index (TLI) = 0.992, Relative Fit Index (RFI) = 0.948, Root Mean Square Error of Approximation (RMSEA) = 0.012, Standardized Root Mean Square Residual (SRMR) = 0.021. All path coefficients were statistically significant. Details are shown in Table 3.

**Table 3:** Path analysis results for the mediation model.

**Outcome variable**	**Predictor variable**	**Effect size (β)**	**95% CI (Lower)**	**95% CI (Upper)**	***p*-value**
**Self-Concept Clarity**	Interoceptive Awareness	0.541	0.491	0.587	^***^
**Athletic Engagement**	Interoceptive Awareness	0.753	0.680	0.824	^***^
	Self-Concept Clarity	0.131	0.061	0.205	^***^
**Future Exercise Possible Self**	Interoceptive Awareness	0.329	0.186	0.469	^***^
	Self-Concept Clarity	0.082	0.013	0.148	^*^
	Athletic Engagement	0.259	0.108	0.408	^**^
**Noticing (MAIA-2)**	Interoceptive Awareness	0.767	0.734	0.799	^***^
**Not-Distracting (MAIA-2)**	Interoceptive Awareness	0.775	0.744	0.803	^***^
**Not-Worrying (MAIA-2)**	Interoceptive Awareness	0.775	0.745	0.804	^***^
**Attention Regulation (MAIA-2)**	Interoceptive Awareness	0.736	0.704	0.767	^***^
**Emotional Awareness (MAIA-2)**	Interoceptive Awareness	0.758	0.726	0.787	^***^
**Self-Regulation (MAIA-2)**	Interoceptive Awareness	0.747	0.712	0.779	^***^
**Trust (MAIA-2)**	Interoceptive Awareness	0.733	0.696	0.767	^***^
**Confidence (AEQ)**	Athletic Engagement	0.728	0.690	0.767	^***^
**Vigor (AEQ)**	Athletic Engagement	0.746	0.711	0.784	^***^
**Dedication (AEQ)**	Athletic Engagement	0.690	0.649	0.728	^***^
**Enthusiasm (AEQ)**	Athletic Engagement	0.711	0.668	0.749	^***^

**Notes.**

* *p* < 0.05.

** *p* < 0.01.

*** *p* < 0.001.

Bootstrap analyses (5,000 resamples) were used to estimate total, direct, and indirect effects. The 95% bias-corrected CIs for all effects did not include zero, confirming statistical significance. Interoceptive awareness had both direct and indirect effects on future exercise possible self, through (a) self-concept clarity alone, (b) athletic engagement alone, and (c) the sequential chain of mediators. Detailed results are presented in Table 4.

**Table 4:** Mediation effect analysis of self-concept clarity and athletic engagement in the association between interoceptive awareness and future exercise possible self.

**Effect type**	**Standardized coefficient**	**Bootstrap 95% CI (Lower)**	**Bootstrap 95% CI (Upper)**	***p*** **-value**	**Relative percentage**
Total effect	0.586	0.538	0.632	^***^	100%
Direct effect	0.329	0.186	0.469	^***^	56.143%
Total indirect effect	0.257	0.140	0.389	^***^	43.857%
Interoceptive awareness → Self-concept clarity → Future exercise possible self	0.044	0.007	0.080	^*^	7.509%
Interoceptive awareness → Athletic engagement → Future exercise possible self	0.195	0.085	0.313	^**^	33.276%
Interoceptive awareness → Self-concept clarity → Athletic engagement → Future exercise possible self	0.018	0.007	0.039	^**^	3.072%

**Notes.**

* *p* < 0.05.

** *p* < 0.01.

*** *p* < 0.001.

Bootstrap confidence intervals are bias-corrected based on 5,000 resamples.

## Discussion

### Summary of key findings

Guided by the embodied cognition framework, this study examined the mechanisms linking interoceptive awareness to adolescents’ future exercise possible self. The key findings were as follows:

(1) Consistent with H1, interoceptive awareness was significantly and positively correlated with adolescents’ future exercise possible self.

(2) Supporting H2, self-concept clarity significantly mediated the association between interoceptive awareness and future exercise possible self.

(3) Supporting H3, athletic engagement significantly mediated the link between interoceptive awareness and future exercise possible self.

(4) Supporting H4, self-concept clarity and athletic engagement jointly formed a significant sequential chain mediation pathway between interoceptive awareness and future exercise possible self.

The results suggest that adolescents’ ability to effectively perceive and interpret internal bodily signals influences both the quality of their self-concept and the positivity of their sports-related experiences, which together strengthen future-oriented exercise self-perceptions. In other words, the future exercise possible self is grounded not only in abstract imagination but also in concrete bodily and emotional experiences. However, the regression coefficient between the body listening dimension of interoceptive awareness and adolescents’ future exercise possible self was not statistically significant. This may be because the body listening dimension tends to reflect past experiences rather than future-oriented expectations (Mehling et al., 2012). Moreover, focusing solely on physical indicators is insufficient to foster a positive future sports outlook. Self-regulation and the interpretation of bodily signals may exert a more significant influence on the development of future sports self-perceptions.

### Theoretical implications and contributions

#### Constructing future exercise self from an embodied cognition perspective

This study is among the first to integrate interoceptive awareness into the framework of future exercise possible self, highlighting the fundamental role of bodily signal processing in the development of future-oriented exercise identities. The findings extend possible self theory by indicating that future self-representations are based not only on cognitive projections but also on ongoing bodily experiences and emotional feedback. This perspective provides a new theoretical lens for understanding how adolescents form stable exercise-related identities in the context of sports.

#### A sequential mechanism from bodily awareness to motivation

The validated cross-sectional chain mediation pathway, that is, interoceptive awareness → self-concept clarity → athletic engagement → future exercise possible self, reveals a layered developmental process from bodily information processing to self-structure integration and finally to motivational experiences. This finding suggests that the key starting point for adolescents’ sports-related psychological development lies in the interpretation of bodily sensations, rather than relying solely on external incentives or cognitive planning. This mechanism may help explain why some adolescents maintain physical activity and develop coherent exercise identities. Notably, while all three proposed mediation pathways were significant, the effect size of athletic engagement accounted for 33.276% of the total effect, whereas self-concept clarity and the chain mediation accounted for only 7.509% and 3.072%, respectively. This indicates that athletic engagement plays a more prominent role in the relationship between interoceptive awareness and future exercise possible self.

### Mechanisms underlying the associations

#### Interoceptive awareness and self-concept clarity

Interoceptive awareness promotes self-concept clarity by helping adolescents detect bodily changes, interpret physiological cues, and integrate these signals into coherent self-schemas. These internal cues provide reliable and repeatable information that enhances the consistency and stability of self-knowledge, including perceptions of physical abilities. When bodily experiences align with personal narratives, adolescents can more easily construct a stable sense of “who I am” and “what I can do” in the context of sports.

#### Interoceptive awareness and athletic engagement

The research findings suggest that athletic engagement is the primary mechanism linking interoceptive awareness to future exercise possible self. This association may be explained by the role of interoceptive awareness in dynamically bridging physiological and psychological states (Wu et al., 2022), which may foster more vigorous, focused, and committed sports participation. Consequently, this engagement enhances positive expectations of future sports self . Previous research has demonstrated that interoceptive awareness affects sports performance through sports perception, decision-making, prediction, and self-regulation (Zeng et al., 2024). This study extends this understanding by clarifying how athletic engagement transforms bodily signals into future-oriented cognitive resources. Positive bodily feedback during physical activity encourages more engaged participation, and the accumulation of such experiences strengthens beliefs in future exercise ability and promotes a positive exercise self-image.

### Comparison with existing literature

The findings are consistent with previous research on adolescent exercise motivation, sports identity formation, mindfulness training, and bodily awareness (Tian & Sun, 2023; Matsuura et al., 2025; Ren et al., 2020; Guillén & Laborde, 2014). However, this study provides new insights:

(1) Bodily experiences are positively correlated with the formation of future-oriented sports identities.

(2) Interoceptive awareness, self-concept clarity, and athletic engagement may enhance the long-term stability of exercise behavior.

These findings confirm that future exercise possible self is rooted in authentic bodily experiences rather than relying solely on external incentives or cognitive planning, providing a more ecologically valid theoretical basis for understanding the persistence and stability of sports participation.

### Practical implications

#### School physical education and curriculum design

Physical education programs can integrate body awareness and emotional regulation practices, such as breath awareness, body scans, and pre-activity emotion identification, to help students better perceive physiological feedback and improve their sense of exercise competence. Encouraging students to express bodily sensations and emotional changes during activity can further enhance interoceptive processing and positive self-evaluation.

#### Psychological interventions for adolescents

In addition to goal-setting and motivational strategies, interventions incorporating mindfulness or interoception-based training (*e.g.*, rhythmic breathing, somatic awareness exercises) can promote self-concept clarity and support sustained physical activity engagement. These programs help adolescents develop a positive cycle of “understanding the body—accepting the self—maintaining participation”.

#### Family and social support

Parents and coaches should focus on adolescents’ bodily experiences and emotional expressions rather than only emphasizing performance outcomes. Creating a family and community environment that values process and experience is more conducive to fostering stable sport identities and positive future exercise orientations.

### Limitations and directions for future research

This study has several limitations:

(1) As a cross-sectional study, it cannot infer causal relationships between variables, nor can it rule out the possibility that there might be other or opposite directional relationships among the variables. Future longitudinal or experimental studies are needed to clarify temporal and developmental mechanisms.

(2) Although Harman’s single-factor test was used to assess common method bias, this method cannot completely eliminate common method variance. Future research should use multiple data sources or objective measurement methods to reduce bias from single self-report data.

(3) Reliance on self-report questionnaires may introduce response bias. Future research could integrate physiological measures (*e.g.*, heart rate variability) or behavioral observations to improve objectivity.

(4) The sample consisted primarily of physically active adolescents. Additionally, the uneven distribution across grades and regions may affect the generalizability of the findings. Future cross-cultural studies and more detailed comparisons across grades and regions are needed to verify the model’s generalizability.

Future research directions include:

(1) Examining the effectiveness of mindfulness or breath-based interventions in improving interoceptive awareness;

(2) Exploring self-concept clarity as a psychological resource for promoting long-term exercise adherence;

(3) Applying machine learning to predict the developmental trajectories of sports identity and provide data for personalized physical activity promotion.

## Conclusion

Based on the embodied cognition framework, this study examined the association between interoceptive awareness and adolescents’ future exercise possible self, and tested the mediating roles of self-concept clarity and athletic engagement. Interoceptive awareness is directly and indirectly related to the prospective exercise self-concept, with direct effects explaining most of the association. Meanwhile, self-concept clarity and athletic engagement serve as critical mediating mechanisms, with athletic engagement playing a significant role. Theoretically, this study integrates interoceptive awareness into the future exercise possible self framework and identifies a continuous pathway from bodily perception to self-knowledge integration and motivational engagement. Practically, the findings suggest that enhancing adolescents’ interoceptive abilities, self-concept clarity, and athletic engagement can foster more positive exercise expectations and identity development.

Overall, the study highlights the interconnected roles of bodily signal interpretation, self-structure, and motivational-emotional resources in adolescents’ sports-related psychological development. Future longitudinal or experimental studies with more diverse samples will help refine and expand this developmental framework.

## Supplemental Information

10.7717/peerj.21780/supp-1 Supplemental Information 1 Raw questionnaire responses assessing interoceptive awareness, self-concept clarity, athlete engagement, and future exercise possible selves among adolescent athletes. anonymized questionnaire responses from adolescents, including demographic variables and item- and total-level scores for interoceptive awareness, self-concept clarity, athletic engagement, and future exercise possible self. All categorical variables are numerically coded and fully explained in the accompanying codebook.

10.7717/peerj.21780/supp-2 Supplemental Information 2 Codebook describing variable definitions and numerical codings used in the study definitions, variable types, numerical codings for categorical variables, Likert-scale response anchors,and total score variables corresponding to the dataset analyzed in this study.

10.7717/peerj.21780/supp-3 Supplemental Information 3 Completed STROBE checklist indicating where each reporting item is addressed in the manuscript

## Additional Information and Declarations

### Data Availability

The following information was supplied regarding data availability: The data is available in the Supplemental Files.
