# Supplementary material for: The association between adolescent interoceptive awareness and future exercise possible self: a mediation model
Source: PeerJ. 2026 Oct 12;14:e21780. doi: 10.7717/peerj.21780 (PMC13671237; doi:10.7717/peerj.21780)
Supplement: Supplementary file 1 [file peerj-14-21780-s002.docx]

**1. Demographic variable coding: Confirmed as follows**

sex: 1 = Male, 2 = Female

grade: 1 = Grade 7; 2 = Grade 8; 3 = Grade 9; 4 = Grade 10; 5 = Grade 11; 6 = Grade 12

region: 1 = North China; 2 = East China; 3 = South China; 4 = Central China; 5 = Southwest China; 6 = Northeast China

residence: 1 = Urban; 2 = Rural / Township

school_type: 1 = Public school; 2 = Private school

sport proficiency level: 1 = first-level athletes; 2 = second-level athletes; 3 = third-level athletes; 4 = non-level athletes (identiffed based on the General Administration of Sports of China; https://www.sport.gov.cn/jts/n5005/c351208/part/211968.pdf)

years of training: 1 =≤1 year ; 2 = 1-2 years; 3 = 3-4 years; 4 = 5-6 years ; 5 =＞6years

**2. Likert-scale response ranges for each instrument: Confirmed as follows**

MAIA-2: 0 = never to 5 = always

SCCS: 1 = strongly disagree to 5 = strongly agree

AEQ: 1 = never to 5 = always

FEPSI: 1 = not at all true of me to 7 = very true of me

**3. Inclusion of total score variables: Yes**

MAIA_total

SCCS_total

AEQ_total

FEPSI_total

**Scale 1. Multidimensional Assessment of Interoceptive Awareness**

| **Variable** | **Item Content** |
| --- | --- |
| MAIA_1 | When I am tense, I notice where the tension is located in my body. |
| MAIA_2 | I notice when I am uncomfortable in my body. |
| MAIA_3 | I notice where in my body I am comfortable. |
| MAIA_4 | I notice changes in my breathing, such as whether it slows down or speeds up. |
| MAIA_5 | I ignore physical tension or discomfort until it becomes more severe. |
| MAIA_6 | I distract myself from sensations of discomfort. |
| MAIA_7 | When I feel pain or discomfort, I try to power through it. |
| MAIA_8 | I try to ignore pain. |
| MAIA_9 | When I feel unpleasant body sensations, I worry about what they might mean. |
| MAIA_10 | I start to worry that something is wrong if I feel any discomfort. |
| MAIA_11 | I can notice an unpleasant body sensation without worrying about it. |
| MAIA_12 | I can stay calm and not worry when I have unpleasant body sensations. |
| MAIA_13 | When I am in discomfort or pain, I can soothe myself. |
| MAIA_14 | I can return awareness to my body if I am distracted. |
| MAIA_15 | I can pay attention to my breath without being distracted by things happening around me. |
| MAIA_16 | I can maintain awareness of my whole body even when a part of me is in pain. |
| MAIA_17 | I trust my body sensations. |
| MAIA_18 | I am at home in my body. |
| MAIA_19 | I feel my body is a safe place. |
| MAIA_20 | I listen for information from my body about my emotional state. |
| MAIA_21 | When I am upset, I take time to explore how my body feels. |
| MAIA_22 | I listen to my body to inform me about what to do. |
| MAIA_23 | I feel that my body sensations help me make decisions. |
| MAIA_24 | I can use my breath to reduce tension. |
| MAIA_25 | When I feel overwhelmed, I can find a calm place inside. |
| MAIA_26 | I can regulate my distress by paying attention to my body sensations. |
| MAIA_27 | I can notice my breath without changing it. |
| MAIA_28 | I can consciously slow down my breathing. |
| MAIA_29 | I feel in control of my breathing. |
| MAIA_30 | I feel connected to my body. |
| MAIA_31 | I feel in touch with my body. |
| MAIA_32 | I feel comfortable being aware of my bodily sensations. |
| MAIA_33 | I enjoy being aware of my bodily sensations. |
| MAIA_34 | I feel at ease in my body. |
| MAIA_35 | I feel confident in my body’s signals. |
| MAIA_36 | I feel that my body supports me. |
| MAIA_37 | I feel my body gives me reliable information. |

**Scale 2. Self-Concept Clarity Scale**

| **Variable** | **Item Content (English)** |
| --- | --- |
| SCC_1 | I have a clear sense of who I am and what I am like. |
| SCC_2 | My beliefs about myself often change. |
| SCC_3 | I spend a lot of time wondering about what kind of person I really am. |
| SCC_4 | Sometimes I feel that I do not really know myself. |
| SCC_5 | My self-image is very stable. |
| SCC_6 | It is hard for me to explain to others what kind of person I am. |
| SCC_7 | My views about myself seem to change depending on the situation. |
| SCC_8 | I seldom experience confusion about who I am. |
| SCC_9 | Even if I wanted to tell someone, I am not sure what I really want. |
| SCC_10 | Even if I wanted to tell others, I do not think I could clearly express what I truly want. |
| SCC_11 | In general, I have a clear sense of who I am. |
| SCC_12 | I usually have difficulty making decisions because I do not really know what I want. |

**Scale 3. Future Exercise Possible Self Inventory**

| **Variable** | **Item Content (English)** |
| --- | --- |
| FEPSI_1 | I will be a good athlete in the future. |
| FEPSI_2 | I will be one of the best athletes on my team. |
| FEPSI_3 | I will perform better in sport than my teammates. |
| FEPSI_4 | My name will appear on the school sports honor roll. |
| FEPSI_5 | I will be a poor athlete. |

**Scale 4. Athlete Engagement Questionnaire**

| **Variable** | **Item Content (English)** |
| --- | --- |
| AEQ_1 | I believe that I can achieve my personal sport goals. |
| AEQ_2 | I am passionate during training and competition. |
| AEQ_3 | I am committed to achieving my sport goals. |
| AEQ_4 | I feel excited about my sport. |
| AEQ_5 | I believe that I can succeed in my sport. |
| AEQ_6 | I feel energetic during training and competition. |
| AEQ_7 | I am determined to achieve my sport goals. |
| AEQ_8 | I am enthusiastic about my sport. |
| AEQ_9 | I believe that I have the technical skills to succeed in sport. |
| AEQ_10 | I feel vigorous during training and competition. |
| AEQ_11 | I am fully engaged in my sport. |
| AEQ_12 | I really enjoy my sport. |
| AEQ_13 | I am confident in my athletic abilities. |
| AEQ_14 | I remain highly focused during training and competition. |
| AEQ_15 | I strive to achieve my sport goals through hard effort. |
| AEQ_16 | I find a lot of enjoyment in my sport. |
