# Supplementary material for: The association between adolescent interoceptive awareness and future exercise possible self: a mediation model
Source: PeerJ. 2026 Oct 12;14:e21780. doi: 10.7717/peerj.21780 (PMC13671237; doi:10.7717/peerj.21780)
Supplement: Supplementary file 2 [file peerj-14-21780-s003.docx]

STROBE Statement—checklist of items that should be included in reports of observational studies

|  | Item No. | Recommendation | Page  No. | Relevant text from manuscript |
| --- | --- | --- | --- | --- |
| **Title and abstract** | 1 | (*a*) Indicate the study's design with a commonly used term in the title or the abstract | 1 | The study design is indicated through the description of a stratified random sampling survey and cross-sectional statistical analyses. |
|  |  | (*b*) Provide in the abstract an informative and balanced summary of what was done and what was found | 1 | The abstract clearly summarizes the study purpose, sample, instruments, analytical methods, main results, and conclusions. |
| Introduction | | | |  |
| Background/rationale | 2 | Explain the scientific background and rationale for the investigation being reported | 2-4 | These sections introduce possible selves theory, interoceptive awareness, self-concept clarity, athletic engagement, and their theoretical integration under embodied cognition. |
| Objectives | 3 | State specific objectives, including any prespecified hypotheses | 4 | The study objectives and four hypotheses (H1–H4) are explicitly stated, including the proposed chain mediation model. |
| Methods | | | |  |
| Study design | 4 | Present key elements of study design early in the paper | 5-6 | The manuscript clearly describes a cross-sectional survey design with mediation modeling and bootstrap estimation. |
| Setting | 5 | Describe the setting, locations, and relevant dates, including periods of recruitment, exposure, follow-up, and data collection | 5 | Data collection time (May 2025), geographic regions (six regions of China), and school grades (Grades 7–12) are clearly specified. |
| Participants | 6 | (*a*) *Cohort study*—Give the eligibility criteria, and the sources and methods of selection of participants. Describe methods of follow-up  *Case-control study*—Give the eligibility criteria, and the sources and methods of case ascertainment and control selection. Give the rationale for the choice of cases and controls  *Cross-sectional study*—Give the eligibility criteria, and the sources and methods of selection of participants | 5-6 | The manuscript reports stratified random sampling, inclusion and exclusion criteria, and the final analytic sample (1,131 valid cases). |
|  |  | (*b*) *Cohort study*—For matched studies, give matching criteria and number of exposed and unexposed  *Case-control study*—For matched studies, give matching criteria and the number of controls per case | N/A | The study did not use a matched design. |
| Variables | 7 | Clearly define all outcomes, exposures, predictors, potential confounders, and effect modifiers. Give diagnostic criteria, if applicable | 6-7 | Exposure: Interoceptive awareness (MAIA-2)  Mediators: Self-concept clarity (SCCS), athletic engagement (AEQ)  Outcome: Future exercise possible self (FEPSI)  Covariates: demographic variables |
| Data sources/ measurement | 8* | For each variable of interest, give sources of data and details of methods of assessment (measurement). Describe comparability of assessment methods if there is more than one group | 6-7 | Each scale is described with item numbers, response formats, reverse scoring, and internal consistency coefficients. |
| Bias | 9 | Describe any efforts to address potential sources of bias | 5,8 | Sampling bias was minimized through stratified random sampling, and common method bias was assessed using Harman’s single-factor test. |
| Study size | 10 | Explain how the study size was arrived at | 5 | The manuscript reports the total number of distributed questionnaires, excluded cases, and final valid sample size, which is adequate for structural equation modeling and multi-regional representation.n-responses. |

Continued on next page

| Quantitative variables | 11 | Explain how quantitative variables were handled in the analyses. If applicable, describe which groupings were chosen and why | 6-7 | All main study variables (interoceptive awareness, self-concept clarity, athletic engagement, and future exercise possible self) were treated as continuous variables and analyzed using their total scores or subscale scores according to standardized scoring procedures. No categorization or arbitrary grouping of continuous variables was performed, in order to preserve statistical power and avoid information loss. |
| --- | --- | --- | --- | --- |
| Statistical methods | 12 | (*a*) Describe all statistical methods, including those used to control for confounding | 7-8 | Descriptive statistics, Pearson correlation analyses, and multiple linear regression analyses were conducted using SPSS 24.0. Structural equation modeling (SEM) with a chain mediation framework was performed in Amos 26.0. Demographic variables (region, sex, grade level, sport proficiency level, years of training, residence, and school type) were included as covariates in regression analyses to control for potential confounding effects. Indirect effects were tested using bias-corrected bootstrap resampling (5,000 resamples) with 95% confidence intervals. |
|  |  | (*b*) Describe any methods used to examine subgroups and interactions | N/A | No subgroup analyses or interaction effects were examined in the present study. |
|  |  | (*c*) Explain how missing data were addressed | 5 | Questionnaires with substantial missing data or invalid response patterns were excluded prior to analysis.  Only complete and valid questionnaires (n = 1,131) were included in the final analyses, and no imputation procedures were applied. |
|  |  | (*d*) *Cohort study*—If applicable, explain how loss to follow-up was addressed  *Case-control study*—If applicable, explain how matching of cases and controls was addressed  *Cross-sectional study*—If applicable, describe analytical methods taking account of sampling strategy | 5,7 | A stratified random sampling strategy was employed, with proportional allocation across regions, sex, grade level, and school type to enhance representativeness. All statistical analyses were conducted on the full analytic sample without weighting, as proportional allocation was controlled during sampling. |
|  |  | (*e*) Describe any sensitivity analyses | N/A | No sensitivity analyses were conducted. |
| Results | | | | |
| Participants | 13* | (a) Report numbers of individuals at each stage of study—eg numbers potentially eligible, examined for eligibility, confirmed eligible, included in the study, completing follow-up, and analysed | 5 | A total of 1,222 questionnaires were distributed. After excluding 91 invalid questionnaires, 1,131 participants were included in the final analyses. |
|  |  | (b) Give reasons for non-participation at each stage | 5 | Invalid questionnaires were excluded due to incomplete responses or failure to meet inclusion criteria. |
|  |  | (c) Consider use of a flow diagram | N/A | A participant flow diagram was not included. |
| Descriptive data | 14* | (a) Give characteristics of study participants (eg demographic, clinical, social) and information on exposures and potential confounders | 5,8 | Participant characteristics including sex, grade level, region, residence, and school type are described. Means and standard deviations of all main study variables are reported in Table 1. |
|  |  | (b) Indicate number of participants with missing data for each variable of interest | 5 | Only complete questionnaires were included in the analyses; therefore, no missing data were present in the analytic sample. |
|  |  | (c) *Cohort study*—Summarise follow-up time (eg, average and total amount) | N/A | Not applicable (cross-sectional study). |
| Outcome data | 15* | *Cohort study*—Report numbers of outcome events or summary measures over time |  | This is a **cross-sectional study**, so there are no outcome events or summary measures reported over time. |
|  |  | *Case-control study—*Report numbers in each exposure category, or summary measures of exposure |  | This is a cross-sectional study, so exposure categories are not reported in this format. |
|  |  | *Cross-sectional study—*Report numbers of outcome events or summary measures | 8-9 | Outcome variables are summarized using means, standard deviations, regression coefficients, and standardized path coefficients from the structural equation model. |
| Main results | 16 | (*a*) Give unadjusted estimates and, if applicable, confounder-adjusted estimates and their precision (eg, 95% confidence interval). Make clear which confounders were adjusted for and why they were included | 8-9 | Regression analyses controlling for demographic covariates are reported with standardized coefficients and significance levels. Direct, indirect, and total effects from SEM are reported with 95% bootstrap confidence intervals. |
|  |  | (*b*) Report category boundaries when continuous variables were categorized | N/A | Continuous variables were not categorized. |
|  |  | (*c*) If relevant, consider translating estimates of relative risk into absolute risk for a meaningful time period | N/A | Relative risk estimates were not applicable in this cross-sectional study. |

Continued on next page

| Other analyses | 17 | Report other analyses done—eg analyses of subgroups and interactions, and sensitivity analyses | N/A | No subgroup analyses, interaction analyses, or sensitivity analyses were conducted in the present study. |
| --- | --- | --- | --- | --- |
| Discussion | | | | |
| Key results | 18 | Summarise key results with reference to study objectives | 9-10 | The key findings are summarized in direct relation to the study objectives and hypotheses, highlighting the positive association between interoceptive awareness and future exercise possible self, as well as the mediating roles of self-concept clarity and athletic engagement, including a significant chain mediation pathway. |
| Limitations | 19 | Discuss limitations of the study, taking into account sources of potential bias or imprecision. Discuss both direction and magnitude of any potential bias | 12 | The manuscript discusses several limitations, including the cross-sectional design (limiting causal inference), reliance on self-report measures (potential reporting bias), and the cultural specificity of the Chinese adolescent sample, with consideration of their potential direction and magnitude. |
| Interpretation | 20 | Give a cautious overall interpretation of results considering objectives, limitations, multiplicity of analyses, results from similar studies, and other relevant evidence | 9-12 | The results are interpreted cautiously within an embodied cognition framework, integrating study objectives, acknowledged limitations, and consistency with existing literature, while avoiding causal overinterpretation. |
| Generalisability | 21 | Discuss the generalisability (external validity) of the study results | 4,12 | The manuscript discusses the external validity of the findings, noting that the large, multi-regional adolescent sample enhances generalisability within China, while also acknowledging the need for cross-cultural validation. |
| Other information | |  | | |
| Funding | 22 | Give the source of funding and the role of the funders for the present study and, if applicable, for the original study on which the present article is based | 13-14 | Funding sources are clearly stated, and the manuscript explicitly notes that the funders had no role in the study design, data collection and analysis, decision to publish, or manuscript preparation. |

*Give information separately for cases and controls in case-control studies and, if applicable, for exposed and unexposed groups in cohort and cross-sectional studies.

**Note:** An Explanation and Elaboration article discusses each checklist item and gives methodological background and published examples of transparent reporting. The STROBE checklist is best used in conjunction with this article (freely available on the Web sites of PLoS Medicine at http://www.plosmedicine.org/, Annals of Internal Medicine at http://www.annals.org/, and Epidemiology at http://www.epidem.com/). Information on the STROBE Initiative is available at www.strobe-statement.org.
